# Is your antiseptic effective against clinical multidrug-resistant microorganisms? A chlorhexidine digluconate formulation demonstrates efficacy even in lower concentrations

**DOI:** 10.1186/2047-2994-4-S1-P34

**Published:** 2015-06-16

**Authors:** NT Mutters, F Günther, S Kaiser, T Fries, U Frank

**Affiliations:** 1 Department of Infectious Diseases, Heidelberg University Hospital, Heidelberg, Germany

## Introduction

Managing Healthcare-associated Infections (HAI) and multidrug-resistant Microorganisms (MDRO) is a daily challenge in hospitals. Universal decolonization by daily bathing with impregnated cloths can result in a reduction of HAIs.

## Objectives

The objective of this study was to measure the effectiveness of a Chlorhexidine digluconate (CHG) formulation against clinical MDRO.

## Methods

Gram-negatives were classified as extensively drug-resistant (XDR-GN) [1]. Additionally, MRSA and VRE isolates were tested. CHG was tested in concentrations of 20mg/ml, 10mg/ml, and 5mg/ml. Two analyses were performed. (1) A quantitative suspension test according to European Standard EN 12353 [2]. Briefly, bactericidal efficacy was determined without organic load, neutralizations was achieved by Caso-bouillon and LTHTh. (2) MIC testing procedures were based on those outlined in the FDA Tentative Final Monograph. Briefly, a 96 well microtitre plate containing doubling dilutions of CHG was set up; broth culltures were standardised to 1x10^8 CFU/mL and added. MIC was defined as the lowest concentration of CHG at which no bacterial growth was observed.

## Results

The suspension tests showed good susceptibility to CHG of all strains. Reduction rates were 99.9-100% for all strains even in lower concentrations. At 15 seconds and a CHG concentration of 5mg/ml a 99.97% reduction of XDR P. aeruginosa, a 99.99% reduction of XRD K. pneumoniae, and a 99.94% reduction of XDR E. coli could be demonstrated. The MIC analysis showed efficacy ranging 19.53 to 39.06 µg/ml in XDR P. aeruginosa, 4.88 to 39.06 µg/ml in XDR K. pneumoniae, and 4.88 to 9.77 µg/ml in XDR E. coli. MRSA showed very low MICs ranging from 1 : 8192 to 1 : 65536; VRE showed MICs ranging from 1 : 512 to 1 : 2048.

## Conclusion

In both analyses, CHG demonstrated an excellent performance against MDRO. The results of these clinical isolates studies and the concentration achieved on patient’s skin demonstrate a very large safety margin when using this formulation. Effective decolonization of patients’ skin can play an important role in reducing risk of HAIs.

## Disclosure of interest

None declared.
